# Socio-economic inequalities in lung cancer mortality in Spain: a nation-wide study using area-based deprivation

**DOI:** 10.1186/s12939-023-01970-y

**Published:** 2023-08-02

**Authors:** Daniel Redondo-Sánchez, Pablo Fernández-Navarro, Miguel Rodríguez-Barranco, Olivier Nuñez, Dafina Petrova, Juan Manuel García-Torrecillas, Jose Juan Jiménez-Moleón, María-José Sánchez

**Affiliations:** 1 Instituto de Investigación Biosanitaria ibs.GRANADA, Granada, 18012 Spain; 2 CIBER of Epidemiology and Public Health (CIBERESP), Madrid, 28029 Spain; 3 Escuela Andaluza de Salud Pública, Granada, 18080 Spain; 4 Cancer and Environmental Epidemiology Unit, National Center for Epidemiology, Carlos III Institute of Health, Madrid, 28029 Spain; 5 Emergency and Research Unit, Torrecárdenas University Hospital, Almería, 04009 Spain; 6 Department of Preventive Medicine and Public Health, University of Granada, Granada, 18071 Spain

**Keywords:** Cancer, Lung cancer, Mortality, Health inequities, Socioeconomic disparities in health

## Abstract

**Background:**

Lung cancer is the main cause of cancer mortality worldwide and in Spain. Several previous studies have documented socio-economic inequalities in lung cancer mortality but these have focused on specific provinces or cities. The goal of this study was to describe lung cancer mortality in Spain by sex as a function of socio-economic deprivation.

**Methods:**

We analysed all registered deaths from lung cancer during the period 2011–2017 in Spain. Mortality data was obtained from the National Institute of Statistics, and socio-economic level was measured with the small-area deprivation index developed by the Spanish Society of Epidemiology, with the census tract of residence at the time of death as the unit of analysis. We computed crude and age-standardized rates per 100,000 inhabitants by sex, deprivation quintile, and type of municipality (rural, semi-rural, urban) considering the 2013 European standard population (ASR-E). We further calculated ASR-E ratios between the most deprived (Q5) and the least deprived (Q1) areas and mapped census tract smoothed standardized lung cancer mortality ratios by sex.

**Results:**

We observed 148,425 lung cancer deaths (80.7% in men), with 73.5 deaths per 100,000 men and 17.1 deaths per 100,000 women. Deaths from lung cancer in men were five times more frequent than in women (ASR-E ratio = 5.3). Women residing in the least deprived areas had higher mortality from lung cancer (ASR-E = 22.2), compared to women residing in the most deprived areas (ASR-E = 13.2), with a clear gradient among the quintiles of deprivation. For men, this pattern was reversed, with the highest mortality occurring in areas of lower socio-economic level (ASR-E = 99.0 in Q5 vs. ASR-E = 86.6 in Q1). These socio-economic inequalities remained fairly stable over time and across urban and rural areas.

**Conclusions:**

Socio-economic status is strongly related to lung cancer mortality, showing opposite patterns in men and women, such that mortality is highest in women residing in the least deprived areas and men residing in the most deprived areas. Systematic surveillance of lung cancer mortality by socio-economic status may facilitate the assessment of public health interventions aimed at mitigating cancer inequalities in Spain.

**Supplementary Information:**

The online version contains supplementary material available at 10.1186/s12939-023-01970-y.

## Introduction

Lung cancer was the main cause of cancer mortality during the year 2020, with an estimated burden of 1.2 million of deaths worldwide [1]. Between 80% and 90% of lung cancer cases are attributable to smoking, whereas the remaining cases are mainly related to carcinogens and environmental exposures, such as radon gas [2].

In Spain, lung cancer is the most deadly cancer in both sexes (1st in men, 3rd in women), with 22,930 deaths in 2020 [1]. The prevalence of smoking in Spain has traditionally been higher in men than in women, although a recent drastic reduction in the gap between the sexes has been observed [3]. During the last decades, and after several legislative measures [4], lung cancer mortality trends in Spain show opposite trends for each sex. While there has been an ascending trend in females, lung cancer mortality in males has been slowly decreasing after 1999, reflecting changes in smoking patterns [5, 6].

Reducing inequalities in cancer outcomes is a main priority of the European Union (EU) [7]. The EU recently created the European Cancer Inequalities Registry to help monitor and reduce cancer inequalities between and within EU Member States. Socio-economic status (SES) is known to play an important role in lung cancer outcomes: people with a lower SES are at higher risk of being diagnosed and dying from lung cancer, and have lower survival [8, 9].

In Europe, research has documented pervasive socio-economic inequalities in lung cancer mortality using either individual-level (e.g., education [10–15]) or area-based measures of SES [16–20]. Previous research suggests that similar socio-economic inequalities may be present in Spain. In particular, in a study of 2.6 million people at risk and 1,067 geocoded lung cancer cases, lower residential SES was associated with an increased risk of developing lung cancer among males but not females [21]. Moreover, when considering all-cause mortality in Spain, women and men residing in the most deprived areas lived 3.2 and 3.8 years less, respectively, than their counterparts residing in the least deprived areas [22].

To the best of our knowledge, in Spain, socio-economic inequalities in lung cancer mortality have only been investigated in specific provinces (e.g., Madrid [23]) or within large cities [16, 20, 24, 25]. For instance, the MEDEA project investigated lung cancer mortality as a function of an area-based deprivation index within 11 large cities [25]. This study reported different socio-economic inequalities for men and women, such that lung cancer mortality was higher for men but lower for women living in the more deprived areas. Similar results have been found in a recent nation-wide study considering socio-economic deprivation on the municipality level [26].

However, no study has examined lung cancer mortality in Spain nation-wide and using a comprehensive, small-area index of socio-economic deprivation. Such a study would provide valuable information on the existing inequalities in a geographically diverse area. The recently developed Spanish Deprivation Index (SDI) allows to characterize socio-economic deprivation in Spain and study its impact on diverse health outcomes [21, 27, 28]. This index uses area-based aggregated information from the national census data from 2011 and provides a standardized assessment of socio-economic deprivation by small areas [29]. In this context, the goal of this study was to describe lung cancer mortality in Spain as a function of socio-economic deprivation. Given the previously documented sex differences in lung cancer trends and outcomes, we aimed to do this separately for men and women.

## Methods

All registered deaths from lung cancer during the period 2011–2017 in Spain were included. Lung cancer mortality data (ICD-10 codes C33-C34 [30]) in Spain during the period 2011–2017 by census tract, year, age and sex were obtained from the National Statistics Institute (protocol agreement BE099-2021). The census tract was defined as the census tract of residence at the time of death.

We used the layer of census tracts of the Spanish Census in 2011. Population data by census tract level, age group, and sex at 1st of January of 2011 were extracted from the National Statistics Institute [31]. Population data were completely merged with the layer of census tracts without losing any information.

To measure SES, we used an area-based deprivation index developed by the Spanish Society of Epidemiology, the Spanish Deprivation Index [29]. The Spanish Deprivation Index was created with data from the Spanish 2011 census conducted by the Spanish Statistical Office, and includes information from six indicators mainly related to employment and education: percentage of employed manual workers, percentage of unemployed manual workers, percentage of employed occasional workers, percentage of unemployed occasional workers, percentage of the population with insufficient education, and percentage of main homes without internet access [29]. The Spanish Deprivation Index was divided in quintiles (Q), where Q1 represents the least deprived areas (highest SES) and Q5 the most deprived areas (lowest SES). Each census tract was assigned with its corresponding SES quintile. The quintiles of the Spanish Deprivation Index by census tract were mapped.

The study protocol has received approval from the Internal Review Board of the Andalusian School of Public Health (CP17/00206), the Granada Provincial Research Review Committee, and the Biomedical Ethics Committee of the Department of Health of the Andalusian Regional Government (study 0072-N-18). The research is in accordance with the principles embodied in the Declaration of Helsinki.

Expected lung cancer deaths for each census tract, sex, and age group were computed by multiplying the census tract population of each sex and age group by the overall age-sex-specific mortality crude rate. For each census tract, expected lung cancer deaths of each age group and sex were added to obtain expected lung cancer deaths by sex. For each sex, census tract smoothed standardized lung cancer mortality ratios (SMR) were computed using a conditional autoregressive model [32] based on fitting a spatial Poisson model with observed deaths as the dependent variable, expected deaths as an offset, and two random effect terms: census tract contiguity and census tract heterogeneity. For the smoothing of the lung cancer mortality ratios, we had to consider neighbouring census tracts. Only 3 census tracts had no neighbouring census tracts, and for these the closest census tract was chosen as an artificial neighbour. Models for each sex were fitted using INLA (Integrated Nested Laplace Approximations for Bayesian inference) with the R-INLA package and a simplified Laplace estimation of the parameters [33, 34].

Observed crude and age-standardized rates were computed for each quintile of deprivation, considering the 2013 European standard population (ASR-E) [35]. Mortality was analysed by SES quintile, sex, age group, and year. Deaths, crude rates and ASR-E were computed by type of municipality (rural: <5,000 inhabitants; semi-rural: ≥5,000 inhabitants and < 25,000 inhabitants; and urban: ≥25,000 inhabitants), sex and SES quintile. Observed age-specific rates were computed. Lung cancer mortality ASR-E ratios (Q5 vs. Q1) were computed by dividing the ASR-E for Q5 by the ASR-E for Q1. Standard error and 95% confidence interval were also computed for the ASR-E ratios adjusting a Poisson model [36].

We used R v.4.2.0 [37] for data management, statistical analysis and mapping. Packages ggplot2 v.3.4.0 [38] and sf v.1.0–9 [39] were used to produce all figures.

## Results

During the period 2011–2017, 151,182 deaths from lung cancer were registered in Spain. Of all these deaths, 1.8% did not merge with the census tract layer and population of 2011, leaving 148,425 lung cancer deaths (80.7% in men) after the merging. Supplementary Fig. 1 shows the yearly distribution of lost deaths, which varies between 1.19% and 2012 and 2.43% in 2017.

**Fig. 1:**
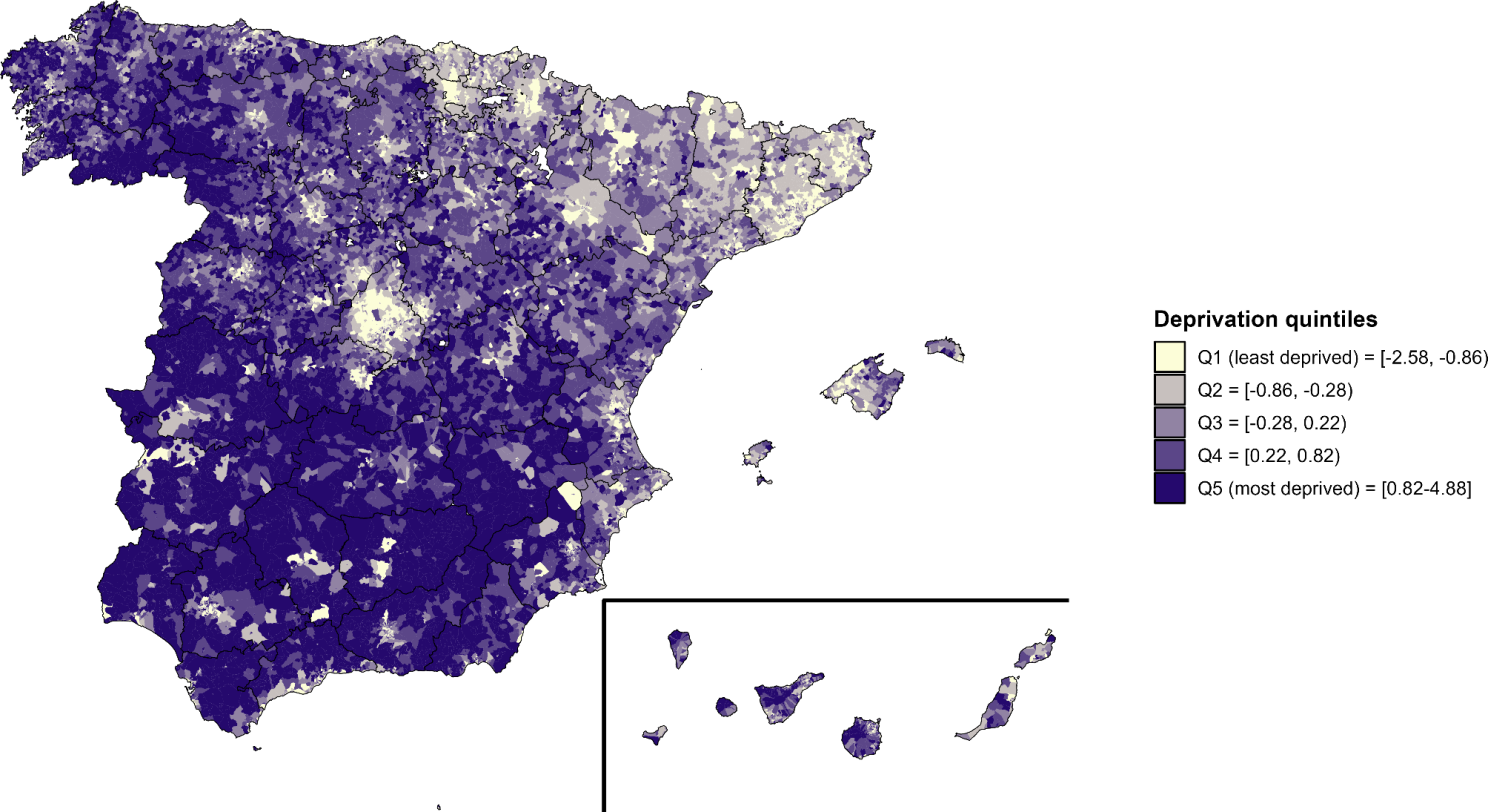
Spanish Deprivation Index quintile by census tract

The spatial distribution by census tract of the Spanish Deprivation Index quintiles is shown in Fig. 1. Overall, there was a north–south pattern, with lower deprivation in the north-east of Spain, Madrid city and its metropolitan area. Higher deprivation was found in southern Spain, the vast majority of the rural areas and the north-west.

**Fig. 2:**
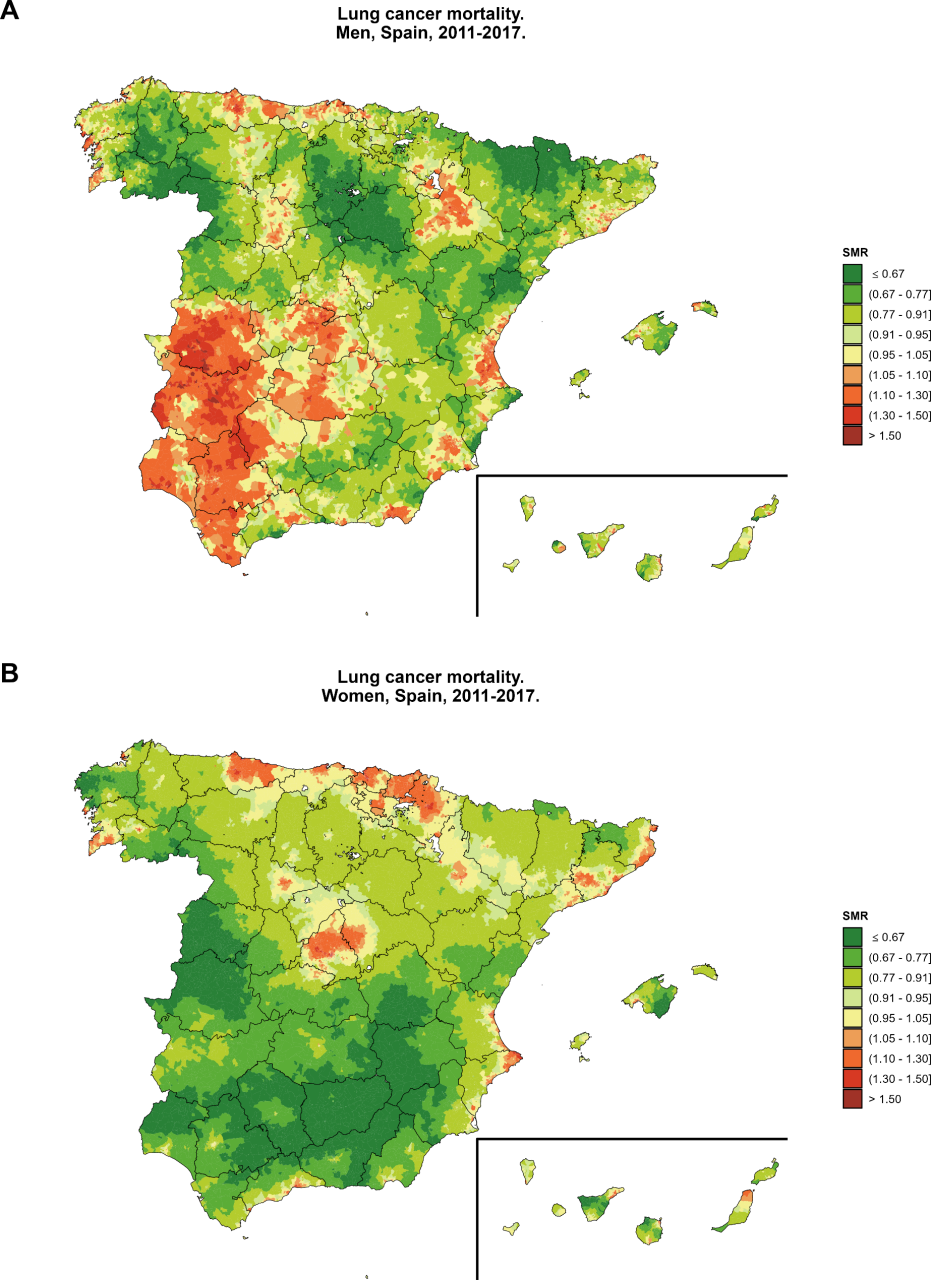
Census tract smoothed standardized lung cancer mortality ratios (SMR) by sex (**A**: men; **B**: women). Spain, 2011–2017

Overall SMR by census tract for Spain, without considering SES, is shown in Fig. 2 for men (Fig. 2A) and women (Fig. 2B). Lung cancer mortality in men was higher in the south-west regions of Spain, Zaragoza, and Valencia. The areas with lower lung cancer mortality in men are in the north of Spain (Huesca, Lleida, Soria, Burgos, Zamora, and Ourense). For women, lung cancer mortality is higher mainly in three areas, one located in Madrid and two in the north of Spain: one in Asturias and another one in Vizcaya, Álava, Guipúzcoa, and Navarra. The lower half of the Peninsula and the north-west have lower lung cancer mortality for women.

Table 1 shows the number of lung cancer deaths, population-year at risk, and lung cancer mortality crude rates and ASR-E per 100,000 inhabitants by sex and SES quintile. 148,425 lung cancer deaths were observed (80.7% in men), distributed in 73.5 deaths per 100,000 men and 17.1 deaths per 100,000 women. Deaths from lung cancer were five times more frequent in men than in women (ASR-E ratio: 5.3).

**Table 1:** Lung cancer mortality in Spain, 2011–2017, by sex and SES quintile. Number of deaths, population-year, crude mortality rate per 100,000 inhabitants, and ASR-E with 95% confidence interval (95% CI) per 100,000 inhabitants. (ASR-E: Age-standardized rates considering the 2013 European standard population)

Sex	SES quintile	Deaths	Population-year	Crude mortality rate	ASR-E (95% CI)
Men	Q1 (least deprived)	22,069	36,841,952	59.9	86.6 (86.2–87.8)
	Q2	24,618	35,503,993	69.3	90.5 (90.1–91.7)
	Q3	25,391	33,345,074	76.1	93.5 (93.0-94.6)
	Q4	24,296	29,861,076	81.4	95.3 (94.8–96.5)
	Q5 (most deprived)	23,409	27,430,214	85.3	99.0 (98.5-100.2)
	**Total**	**119,783**	**162,982,309**	**73.5**	**92.6** **(92.4–93.1)**
Women	Q1 (least deprived)	7,918	39,652,172	20.0	22.2 (21.7–22.7)
	Q2	6,495	36,887,942	17.6	18.3 (17.9–18.7)
	Q3	5,798	34,002,808	17.1	17.0 (16.6–17.4)
	Q4	4,719	29,913,912	15.8	15.1 (14.7–15.6)
	Q5 (most deprived)	3,712	26,894,308	13.8	13.2 (12.8–13.7)
	**Total**	**28,642**	**167,351,142**	**17.1**	**17.4** **(17.2–17.6)**

Women residing in the most affluent areas had higher mortality from lung cancer (Table 1), compared to women residing in the most deprived areas, with a clear gradient among the different quintiles of deprivation (ASR-E: 22.2 in Q1–18.3 in Q2–17.0 in Q3–15.1 in Q4–13.2 in Q5). The pattern was completely reversed for men (Table 1), where the highest lung cancer mortality occurred in men residing in the most deprived areas: ASR-E for least deprived areas (Q1) and most deprived areas (Q5), respectively, were 86.6 and 99.0 per 100,000 men.

The consistency of the socio-economic inequalities found was supported by a sensitivity analysis using percentiles instead of quintiles to add more detail (Supplementary Fig. 2). When using percentiles, the mortality gradient was still clear in both sexes, with especially noticeable ASR-Es in the extremes of the SES distribution. For men, the highest standardized rate was obtained for men residing in P100 and P99 areas (lowest SES), with 121.9 and 110.7 deaths per 100,000 men respectively, while the lowest ASR-E was found in P1 and P2 areas (highest SES), with 78.9 and 80.2 deaths per 100,000 men respectively. In women, the lowest standardized rate was obtained for women residing in P92 areas, with 10.8 deaths per 100,000 women, whereas the highest ASR-E was found in P1 areas (highest SES), with 27.1 deaths per 100,000 women.

Considering the type of municipality, the lung cancer mortality ASR-E was higher in urban areas in both men and women of all quintiles of deprivation (Supplementary Tables 1, Supplementary Fig. 3). In men, we found a direct association between deprivation and lung cancer mortality in all types of municipalities, with higher mortality in urban and semi-rural areas. In women this association was reversed, and no clear distinction was found between rural and semi-rural areas.

**Fig. 3:**
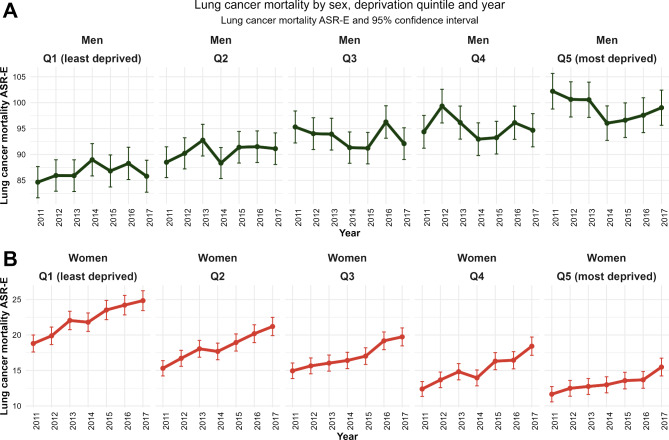
Lung cancer mortality by sex, deprivation quintile, and year. Lung cancer mortality ASR-E per 100,000 inhabitants and 95% confidence interval. Spain, 2011–2017. (ASR-E: Age-standardized rates considering the 2013 European standard population)

In addition, the existing socio-economic inequalities in lung cancer mortality were consistent over time. Figure 3 shows lung cancer mortality by sex, deprivation quintile, and year. An upward trend over time is observed for women residing in all areas (Fig. 3B), with the mortality rate being lower in most deprived areas. For women residing in the least deprived areas (Q1), the ASR-E per 100,000 women went from 18.8 to 2011 to 24.8 in 2017, whereas for women residing in the most deprived areas (Q5) the ASR-E per 100,000 women in 2011 was 11.7, compared to 15.5 in 2017. The time trend in men (Fig. 3A) is not clear, but there are relevant differences between deprivation quintiles.

In Table 2 and Supplementary Fig. 4 we show the lung cancer mortality ratio (ASR-E in Q5 divided by ASR-E in Q1) for each sex and year, demonstrating minor changes over time in men and women. For the whole period, men residing in the most deprived areas (Q5) had between 8% (in 2014) and 21% (in 2011) more lung cancer deaths than men residing in the most affluent areas (Q1). On the contrary, women residing in most affluent areas (Q1) had between 79% (2016) and 59% (2012) more lung cancer deaths than women residing in the most deprived areas (Q5). The evolution of the mortality ratio is relatively stable for both sexes for the whole period 2011–2017, suggesting that socio-economic differences by sex in lung cancer mortality are not widening or narrowing over time.

**Table 2:** Lung cancer mortality ASR-E ratio (Q5 vs. Q1) and 95% confidence interval by sex and year. Spain, 2011–2017. (ASR-E: Age-standardized rates considering the 2013 European standard population)

Sex	Year	ASR-E mortality ratio Q5 vs. Q1 (CI 95%)
Men	2011	1.21 (1.15–1.27)
	2012	1.17 (1.12–1.23)
	2013	1.17 (1.11–1.23)
	2014	1.08 (1.03–1.13)
	2015	1.11 (1.06–1.17)
	2016	1.11 (1.05–1.16)
	2017	1.15 (1.10–1.21)
Women	2011	0.62 (0.56–0.69)
	2012	0.63 (0.57–0.70)
	2013	0.58 (0.52–0.64)
	2014	0.60 (0.54–0.66)
	2015	0.58 (0.52–0.64)
	2016	0.56 (0.51–0.62)
	2017	0.62 (0.57–0.69)

**Fig. 4:**
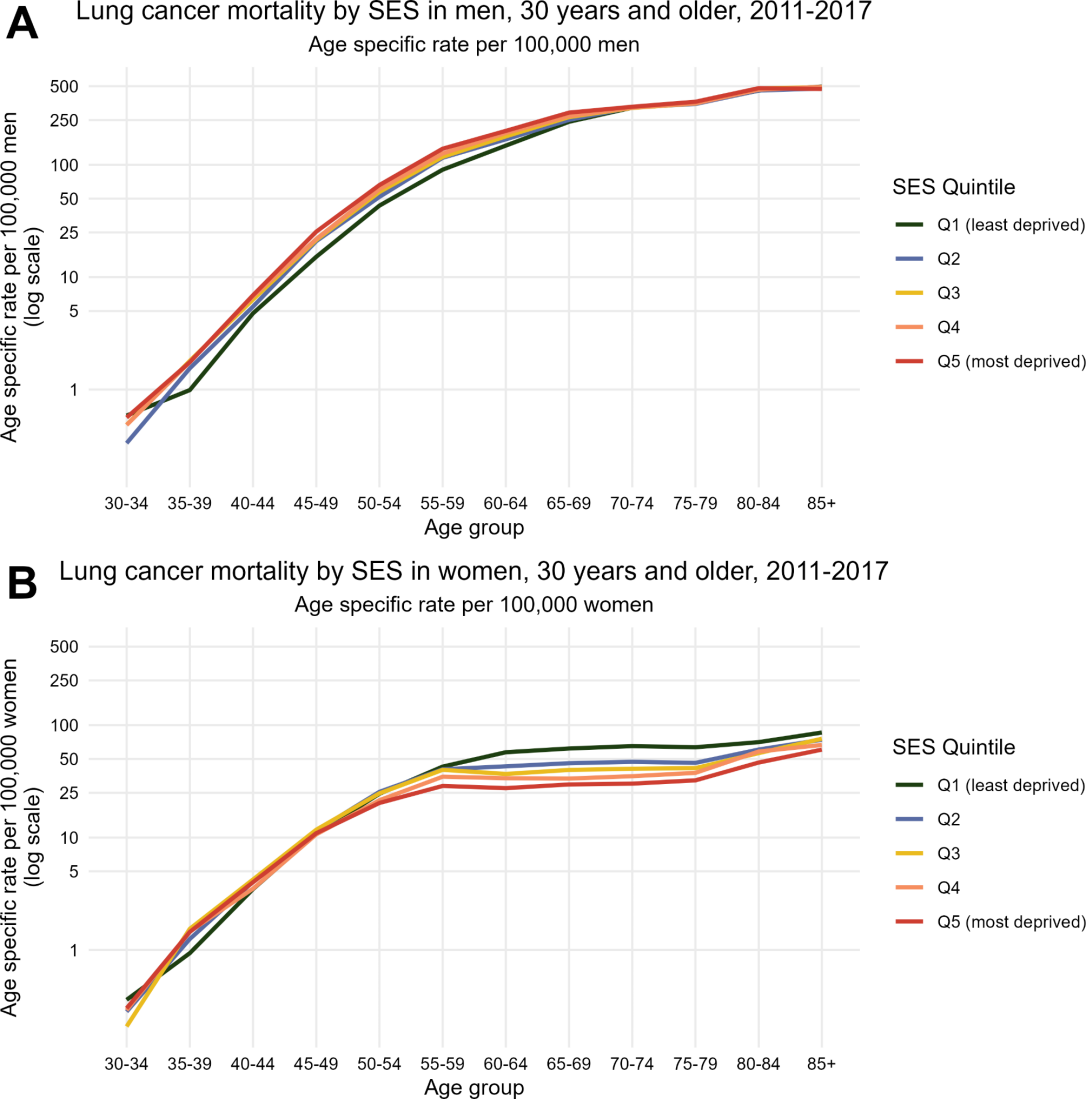
Age-specific lung cancer mortality rate by sex (**A**: men, **B**: women) for persons ≥ 30 years old. Spain, 2011–2017. Age specific rate per 100,000 inhabitants

The overall pattern of socio-economic inequalities by sex is also observed when analysing lung cancer mortality by SES quintile and age group using age-specific rates (Fig. 4). The mortality difference is detected from 50 years on for women (Fig. 4B), and between 35 and 69 years in men (Fig. 4A), with a gradient between the five quintiles of SES in both sexes.

## Discussion

Overall, we found that lung cancer mortality varies strongly as a function of area-based deprivation, with opposite effects in men and women. Lung cancer mortality was highest among men residing in the most deprived areas and among women residing in the least deprived areas.

The documented socio-economic inequalities in men are in agreement with previous research in different European countries using diverse individual and area-based indicators of SES [11–13, 15–18, 20, 23–26, 40–47]. In contrast, the association of SES with lung cancer mortality among women is less consistent and seems to be region-dependent. In particular, higher lung cancer mortality is consistently found for women of lower SES in Western and Northern European countries regardless of the SES measure used [13, 17, 18, 43]. The opposite effect – higher mortality among women with higher SES - has been found in Italy [11] and Spain [25, 26, 47]. In fact, several previous studies considering different European cities and regions have found this divergent pattern for these Mediterranean regions [10, 14, 16]. Given the time lag between smoking uptake and the occurrence of lung cancer (20–50 years depending on smoking intensity), these results are thought to reflect changes in smoking patterns [14, 26]. For instance, in Spain, smoking was more prevalent among men than women before the 1970s, after which smoking initiation started to be common among highly educated women, with less-educated women following suit in the 1980s [26]. As a result, Spain has experienced one of the largest increases in lung cancer mortality among women worldwide [48].

To look at more recent smoking trends, we used data from the Spanish National Health Survey [49], a large periodic representative survey of adults ≥ 15 years old residing in Spain. This survey is the main data source for the prevalence of smoking in Spain. The survey also records, since 1997, the individual educational level of respondents, one of the main components of the deprivation index used in this study. In Supplementary Fig. 5 we show the prevalence of smoking (in percentage) by sex, educational level, and year of the survey. In the last two decades, smoking prevalence has been declining over time in both men and women, and more strongly among the groups with higher education level. The decline in smoking prevalence is much less pronounced in men with low educational level, which may suggest that the higher lung cancer mortality in men residing in the most deprived areas may persist. The higher mortality in women residing in the least deprived areas could be partially due to the historically higher smoking prevalence in women with high educational level (university studies or secondary education). Because the prevalence in these groups has been declining steeply, we would expect socio-economic differences in lung cancer mortality for women to decrease in the following years. In fact, in a previous study on cancer incidence by SES in Spain, we found that lower SES was associated with an increased risk of lung cancer incidence among males, but no relationship was found for females [21].

Overall, with tobacco smoking being the main risk factor for lung cancer incidence, and the poor prognosis of the disease (5-year net survival in Spain: 13.2% [50]), differences in tobacco consumption patterns by SES seem to be the underlying cause of the inequalities found in mortality. However, other factors such as financial issues, access to health services, or marginalization can affect lung cancer mortality [7].

The mapping of the SMR in our study shows a very similar pattern as other recent work for Spain and Portugal [51], with the present study considering a more recent period of mortality (2011–2017 instead of 2003–2012) and having a smaller unit of analysis (35,960 census tracts instead of 8,097 municipalities), characteristics that can produce stronger relationships between SES and cancer outcomes [52, 53].

The socio-economic inequalities in lung cancer mortality remained fairly stable over time and across urban and rural areas. The higher lung cancer mortality we found in urban areas is in accordance with a previous research conducted in the Madrid Autonomous Region [23], where after adjusting for deprivation quintile and air pollution, rural areas had a rate ratio of lung cancer mortality of 0.73 in men aged < 65 years, 0.84 in men aged ≥ 65 years, 0.51 in women aged < 65 years and 0.66 in women aged ≥ 65 years, considering the city of Madrid (urban area) as reference.

The overall lung cancer mortality rates of our study were aligned with the information provided for Spain in the Global Cancer Observatory (GCO) [1] and the European Cancer Information System (ECIS) [54]. Following the lung cancer mortality trends in Spain, which are increasing in females and decreasing in males [6], the ASR-E obtained in our study (2011–2017) were 92.6 and 17.4 deaths per 100,000 inhabitants in males and females respectively, which are consistent with the estimation of ECIS for 2020: 80.8 deaths per 100,000 males and 21.3 deaths per 100,000 females [54]. For comparison with the GCO data, we computed the age-standardized rates considering the World standard population (ASR-W) [55]. For Spain during the period 2011–2017, ASR-W were 38.8 and 8.6 deaths per 100,000 inhabitants for males and females respectively, in accordance with the estimation of GCO of 33.2 deaths per 100,000 males and 10.4 per 100,000 females [1].

One limitation of this study is that the deprivation index and population data are referred to 2011, whereas the mortality data covers a longer period (2011–2017). Deprivation may have changed during 2011–2017, nevertheless we argue that aggregated measures of socio-economic inequalities are likely to be fairly consistent over time. Moreover, the use of the European Index of Deprivation [56] could have facilitated the replication and comparison of the present study in the European context, but unfortunately this index is not updated for 2011 in Spain.

Whereas the deprivation index is not based on direct information about income, in a previous study we discovered that, at the census tract level, this deprivation index and the average income per person are correlated in Spain [21]. An additional constraint of the research is that a small percentage of lung cancer deaths (less than 2% overall) were lost due to changes in the cartography over time. The loss is higher for the last years of the period 2011–2017 (Supplementary Fig. 1), especially after 2013, but there are no reasons to believe this is uneven across the levels of SES. Unfortunately, geocoded deaths or a list of changes over time of the different census tract layers are not available.

One of the main strengths of the study is the use of exhaustive mortality data collected by an official institution. Although some issues with the coding of the cause of death are plausible [57, 58], for lung cancer this is not as likely as in other anatomical locations such as uterus [59, 60]. Finally, the use of nation-wide mortality data covering several years allowed us to describe socio-economic inequalities in a large geographically diverse area.

More broadly, the current results show the need for interventions targeting the social determinants of health, accompanied by regular monitoring over time using standardized measures to better understand the effects of policies on lung cancer mortality. Forthcoming interventions need to consider SES as one of the main components to maximize the effect produced. For instance, Europe’s Beating Cancer Plan promotes the implementation of lung cancer screening with low-dose computed tomography in the European Union [61]. In Spain, multiple scientific societies are piloting the CASSANDRA project (Cancer Screening, Smoking Cessation and Respiratory Assessment), aiming to assess the feasibility of population screening through recruitment of patients in more than 20 public, private, and primary care centres. Previous works suggest that novel interventions such as lung cancer screening can widen the existing mortality inequalities [62–65]. A recent systematic review [62] found that, in the United States, lower household income was linked with lower screening eligibility [63], and lower income patients were less likely to complete screening or have the intention to be screened [64, 65]. A key premise behind the success of lung cancer screening is the identification of high-risk profiles (e.g. heavy smokers or ex-smokers who used to smoke heavily). The current results suggest that SES should be evaluated as another relevant variable to include in profiling.

A complex approach involving both individual-level and population-level interventions is needed to reduce the extent of the socio-economic inequalities we found in our study. Improving access to healthcare in lower socio-economic groups (specially in men) and reducing the exposure to risk factors like smoking or environmental pollutants could reduce the inequalities in lung cancer mortality, while more knowledge about the root causes is required for such interventions to be more effective.

## Conclusions

Socio-economic status is strongly related to lung cancer mortality, showing opposite patterns in men and women, such that mortality is highest in women residing in the least deprived areas and men residing in the most deprived areas. Documenting socio-economic inequalities in lung cancer mortality could help direct policies and interventions aiming to reduce inequalities. Systematic surveillance of lung cancer mortality by socio-economic status may facilitate the assessment of public health interventions aimed at mitigating cancer health inequalities in Spain.

## Electronic supplementary material

Below is the link to the electronic supplementary material.

Supplementary Material 1

Supplementary Material 2

## Data Availability

Population datasets and cartography are available publicly at the Spanish National Statistics Institute webpage (https://www.ine.es/en/index.htm). Mortality datasets analysed during the current study are not accessible to the public as they are subject to confidentiality restrictions. However, they can be obtained from the Spanish National Institute of Statistics on reasonable request.

## List of abbreviations

ASR-E: Age-standardized rates considering the 2013 European standard population
ASR-W: Age-standardized rates considering the World standard population
ECIS: European Cancer Information System
GCO: Global Cancer Observatory
SES: Socio-economic status
SMR: Census tract smoothed standardized lung cancer mortality ratios

## Declarations

### Disclaimer

This article presents an independent research. The views expressed are those of the authors, and not necessarily those of the Carlos III Institute of Health.
