# Supplementary material for: Socio-economic inequalities in lung cancer mortality in Spain: a nation-wide study using area-based deprivation
Source: Int J Equity Health. 2023 Aug 2;22:145. doi: 10.1186/s12939-023-01970-y (PMC10399030; doi:10.1186/s12939-023-01970-y)

Supplementary Figure 1. Percentage (%) and number of lost lung cancer deaths (N) by year, after the merging of mortality with population and cartography. Spain, 2011-2017.

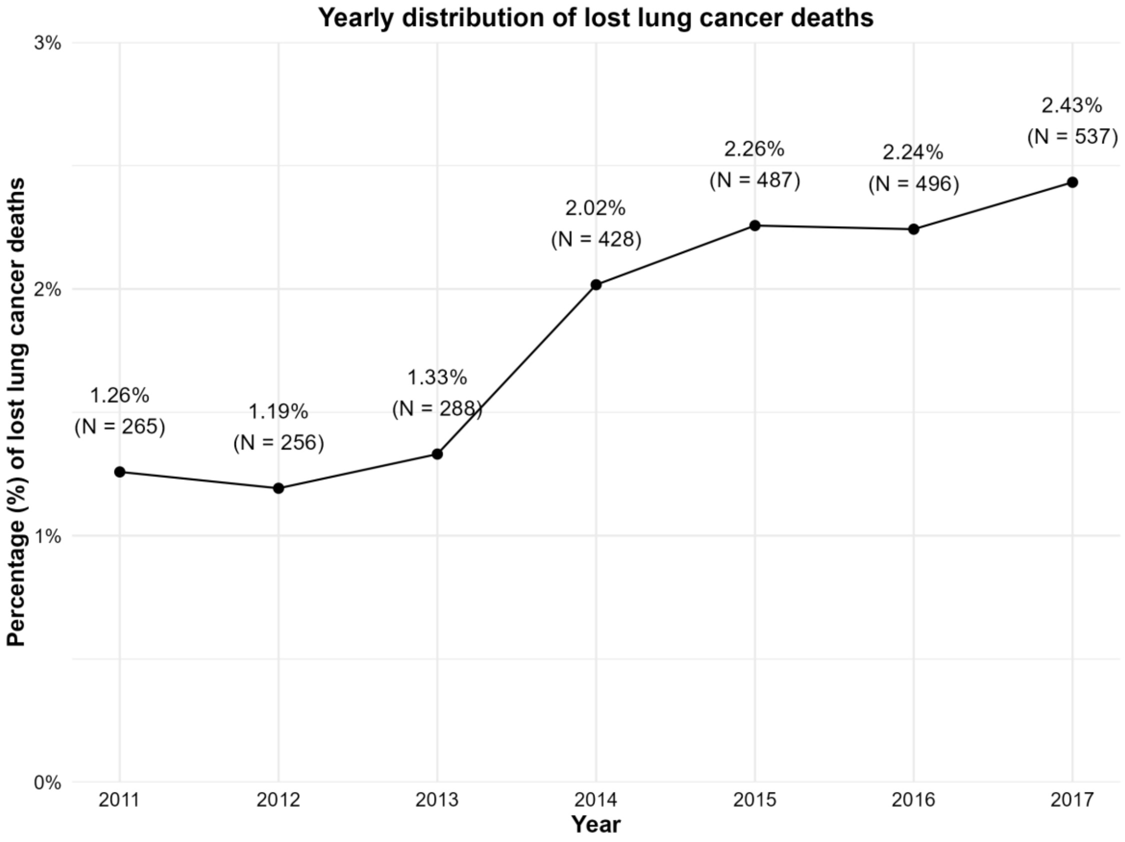

Supplementary Figure 2. Lung cancer mortality by sex and SES percentile. Spain, 2011-2017. Age-standardized lung cancer mortality rate per 100,000 inhabitants. (ASR-E: Age-standardized rates considering the 2013 European standard population).

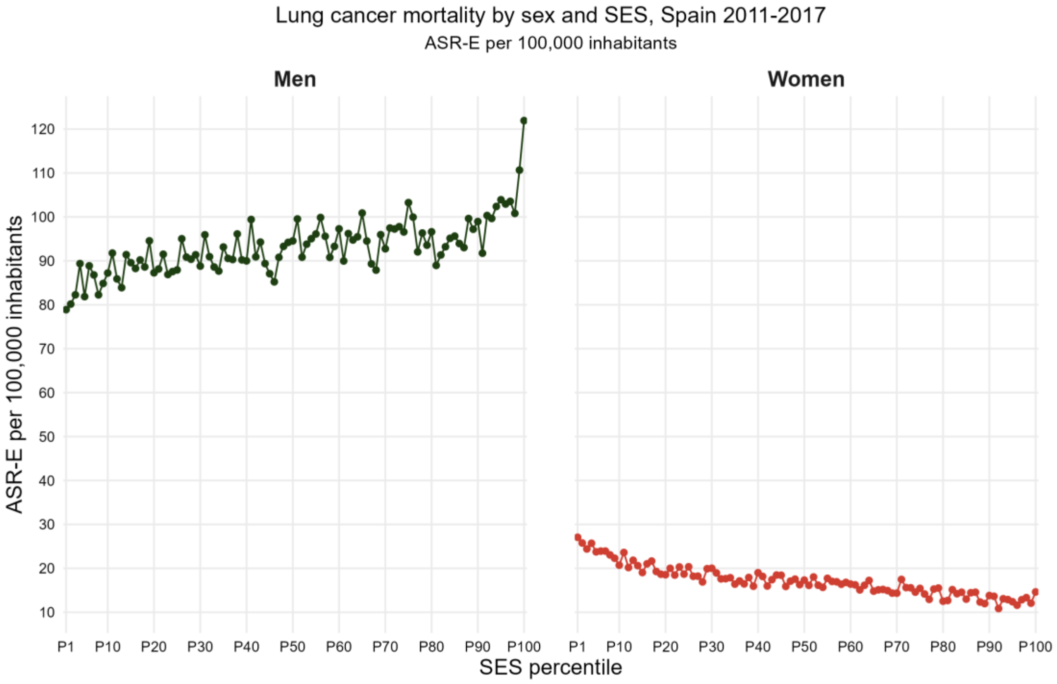

Supplementary Figure 3. Lung cancer mortality by type of municipality (urban, semi-rural, and rural), sex, and deprivation quintile. Lung cancer mortality ASR-E per 100,000 inhabitants and 95% confidence interval. Spain, 2011-2017. (ASR-E: Age-standardized rates considering the 2013 European standard population).

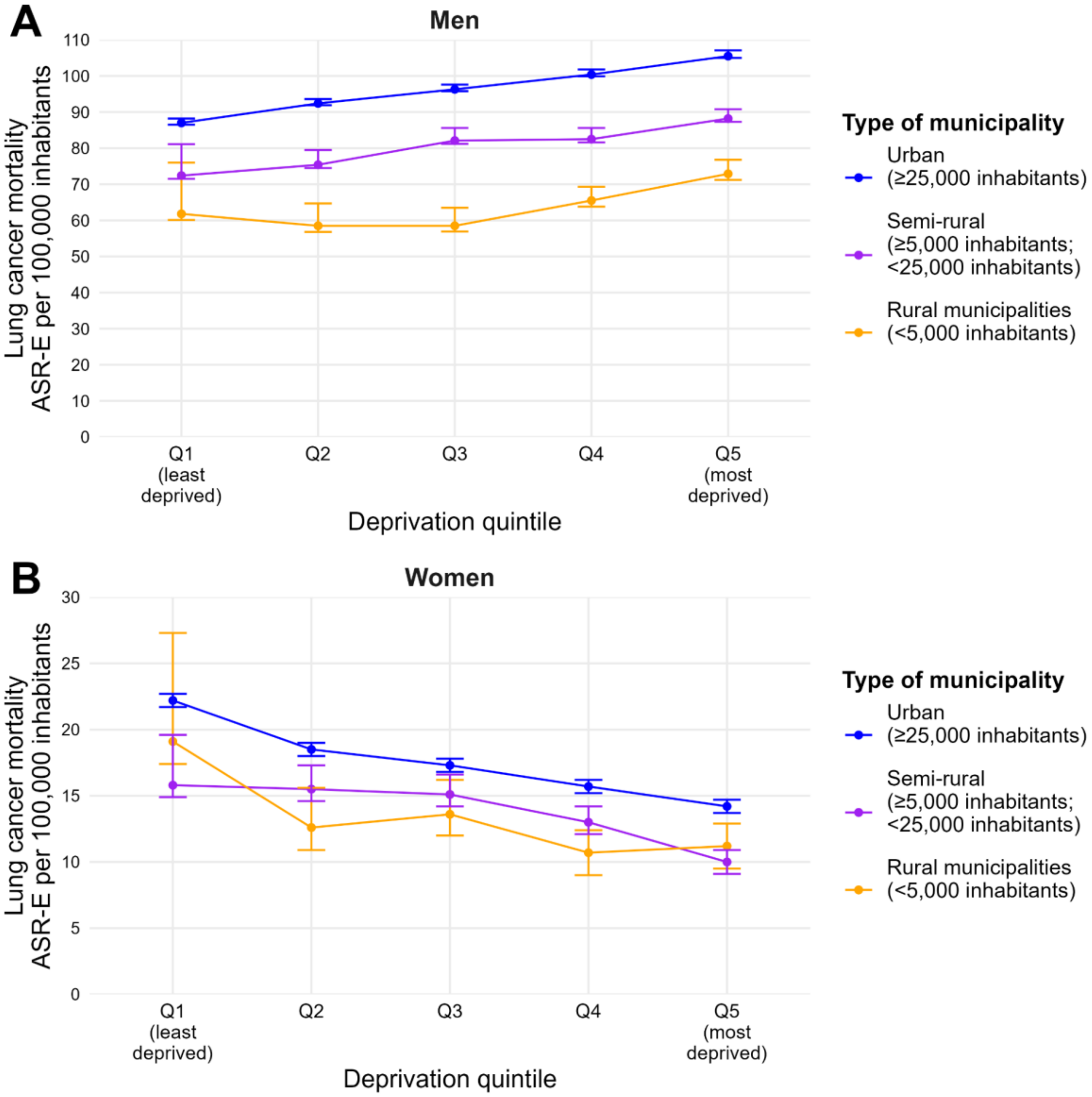

Supplementary Figure 4. Lung cancer mortality ratio (Q5: most deprived vs Q1: least deprived) by sex and year. Spain, 2011-2017. (ASR-E: Age-standardized rates considering the 2013 European standard population).

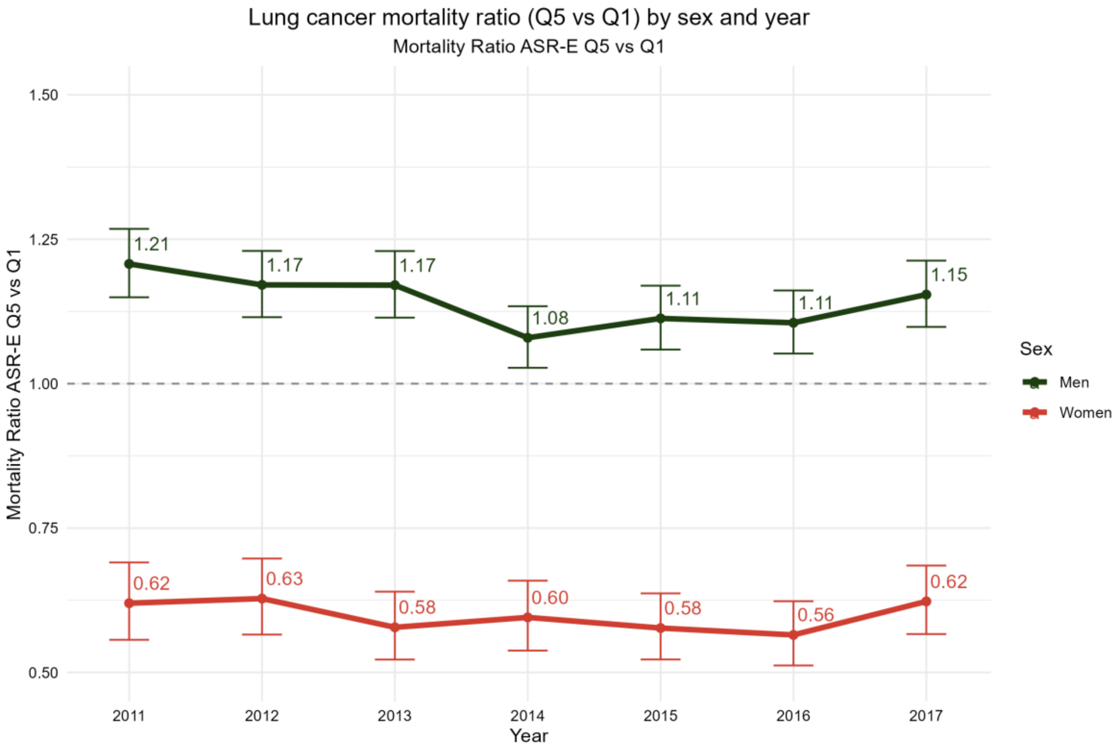

Supplementary Figure 5. Smoking prevalence (%) by sex, educational level and year, 1997-2020. Source: Spanish National Health Survey.

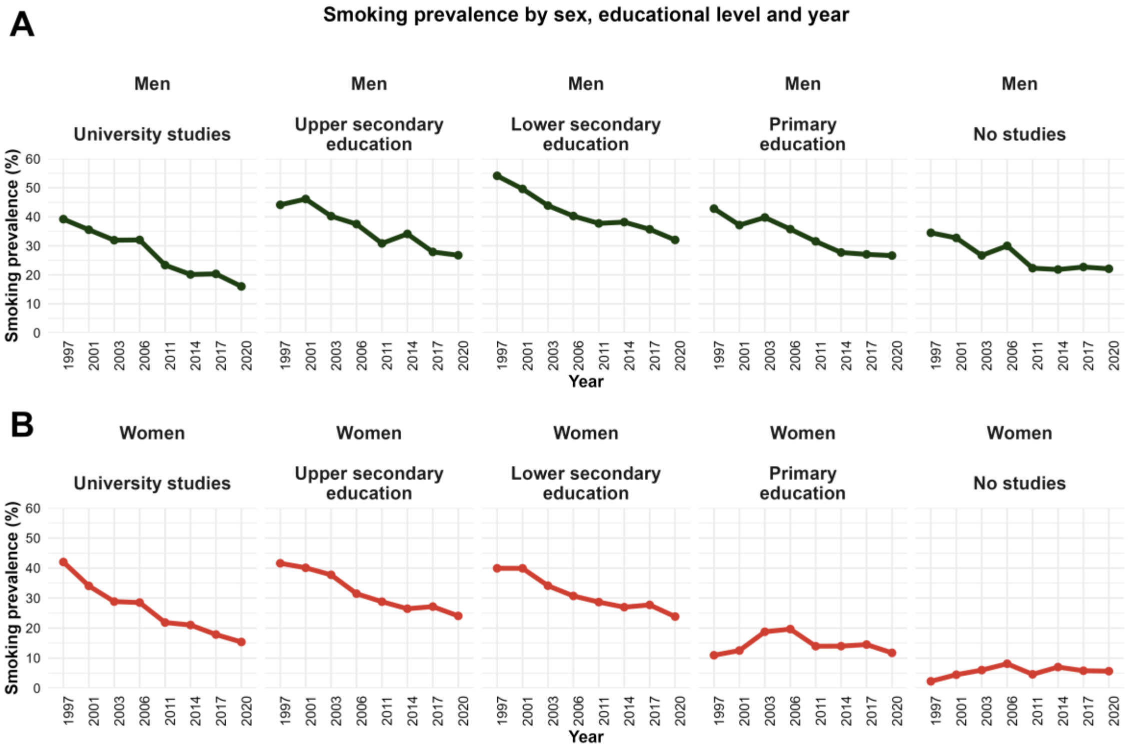

Supplement: Supplementary file 1 — Supplementary Material 1 [file 12939_2023_1970_MOESM1_ESM.pdf]
