# Supplementary material for: Socio-economic inequalities in lung cancer mortality in Spain: a nation-wide study using area-based deprivation
Source: Int J Equity Health. 2023 Aug 2;22:145. doi: 10.1186/s12939-023-01970-y (PMC10399030; doi:10.1186/s12939-023-01970-y)
Supplement: Supplementary file 2 — Supplementary Material 2 [file 12939_2023_1970_MOESM2_ESM.pdf]

Supplementary Table 1. Lung cancer mortality in Spain, 2011-2017, by type of municipality (rural, semi-rural, or urban), sex and SES quintile. Number of deaths, population-year, crude mortality rate per 100,000 inhabitants and ASR-E with 95% confidence interval (95% CI) per 100,000 inhabitants. (ASR-E: Age-standardized rates considering the 2013 European standard population).

Rural / Semi-rural / Urban	Sex	SES quintile	Deaths	Population-year	Crude mortality rate	ASR-E (95% CI)
**Rural municipalities (<5,000 inhabitants)**	**Men**	Q1 (least deprived)	74	124,985	59.2	61.8 (60.1-76)
		Q2	344	534,219	64.4	58.5 (56.8-64.7)
		Q3	567	754,306	75.2	58.5 (56.9-63.5)
		Q4	1,218	1,279,698	95.2	65.5 (63.8-69.3)
		Q5 (most deprived)	1,446	1,343,601	107.6	72.9 (71.2-76.8)
		**Total**	**3,649**	**4,036,809**	**90.4**	**65.9 (64.1-68.1)**
	**Women**	Q1 (least deprived)	22	111,174	19.8	19.1 (17.4-27.3)
		Q2	74	474,586	15.6	12.6 (10.9-15.6)
		Q3	132	661,241	20.0	13.6 (12-16.2)
		Q4	190	1,125,950	16.9	10.7 (9-12.4)
		Q5 (most deprived)	226	1,213,569	18.6	11.2 (9.5-12.9)
		**Total**	**644**	**3,586,520**	18.0	**11.8 (10.8-12.8)**
**Semi-rural (≥5,000 inhabitants; <25,000 inhabitants)**	**Men**	Q1 (least deprived)	278	595,399	46.7	72.4 (71.5-81.1)
		Q2	1,309	2,082,962	62.8	75.4 (74.5-79.5)
		Q3	2,105	2,583,343	81.5	82.1 (81.2-85.6)
		Q4	2,806	3,081,085	91.1	82.5 (81.6-85.6)
		Q5 (most deprived)	4,391	4,433,037	99.1	88.2 (87.3-90.8)
		**Total**	**10,889**	**12,775,826**	**85.2**	**83.2 (82.6-84.7)**
	**Women**	Q1 (least deprived)	71	571,858	12.4	15.8 (14.9-19.6)
		Q2	309	1,986,222	15.6	15.5 (14.6-17.3)
		Q3	427	2,477,839	17.2	15.1 (14.2-16.6)
		Q4	498	2,942,268	16.9	13 (12.1-14.2)
		Q5 (most deprived)	561	4,295,760	13.1	10 (9.1-10.9)
		**Total**	**1,866**	**12,273,947**	**15.2**	**12.7 (12.1-13.3)**
**Urban (≥25,000 inhabitants)**	**Men**	Q1 (least deprived)	21,717	36,121,568	60.1	87 (86.5-88.2)
		Q2	22,965	32,886,812	69.8	92.4 (91.9-93.6)
		Q3	22,719	30,007,425	75.7	96.3 (95.8-97.6)
		Q4	20,272	25,500,293	79.5	100.4 (99.9-101.8)
		Q5 (most deprived)	17,572	21,653,576	81.2	105.5 (105-107.1)
		**Total**	**105,245**	**146,169,674**	**72.0**	**95.3 (95.1-95.9)**
	**Women**	Q1 (least deprived)	7,825	38,969,140	20.1	22.2 (21.7-22.7)
		Q2	6,112	34,427,134	17.8	18.5 (18-19)
		Q3	5,239	30,863,728	17.0	17.3 (16.8-17.8)
		Q4	4,031	25,845,694	15.6	15.7 (15.2-16.2)
		Q5 (most deprived)	2,925	21,384,979	13.7	14.2 (13.7-14.7)
		**Total**	**26,132**	**151,490,675**	**17.2**	**18.0 (17.8-18.2)**
